# Assessment of mesenchymal stem/stromal cell-based therapy in K/BxN serum transfer-induced arthritis

**DOI:** 10.3389/fimmu.2022.943293

**Published:** 2022-10-10

**Authors:** Mercedes Lopez-Santalla, Carmen Conde, Angela Rodriguez-Trillo, Marina I. Garin

**Affiliations:** ^1^ Division of Hematopoietic Innovative Therapies, Biomedical Innovation Unit, Centro de Investigaciones Energéticas Medioambientales y Tecnológicas (CIEMAT), Madrid, Spain; ^2^ Centre for Biomedical Network Research on Rare Diseases (CIBER-ER) and Advanced Therapy Unit, Madrid, Spain; ^3^ Advanced Therapy Unit, Health Research Institute- Fundación Jiménez Díaz, University Hospital, Universidad Autónoma de Madrid (IIS-FJD, UAM), Madrid, Spain; ^4^ Laboratorio de Reumatología Experimental y Observacional, Instituto de Investigación Sanitaria de Santiago (IDIS), Hospital Clínico Universitario de Santiago de Compostela (CHUS), Servicio Gallego de Salud (SERGAS), Santiago de Compostela, Spain

**Keywords:** Mesenchymal stem/stromal cell-based therapy, K/BxN serum transfer-induced arthritis, Joint inflammation, cell therapy, Immunomodulation

## Abstract

Rheumatoid arthritis (RA) is an autoimmune disease characterized by synovial hyperplasia and cartilage/bone destruction with systemic comorbidities. Despite advances in understanding the aetiology of RA and novel biologic drugs, a substantial number of individuals with RA remain intolerant or resistant to these therapies. In this context, mesenchymal stem/stromal cell (MSC)-based therapy has emerged as an innovative therapeutic alternative to address unresolved treatment issues for patients with RA thanks to the immunomodulatory properties of these cells. The majority of preclinical studies in MSC-based therapy have been conducted using the well-known collagen-induced arthritis (CIA) mouse model however due to its low incidence, the mouse strain restriction and the prolonged induction phase of collagen-induced arthritis, alternative experimental models of RA have been developed such as K/BxN serum transfer-induced arthritis (STIA), which mimics many of human RA features. In this study, we evaluate whether the K/BxN STIA model could be used as an alternative model to study the immunomodulatory potential of MSC-based therapy. Unexpectedly, our data suggest that adipose-derived MSC-based therapy is unsuitable for modulating the progression of K/BxN serum-transfer arthritis in mice despite the various experimental parameters tested. Based on the differences in the immune status and monocytic/macrophage balance among the different arthritic models, these results could help to identify the cellular targets of the MSCs and, most importantly to predict the RA patients that will respond positively to MSC-based therapy.

## Introduction

Rheumatoid arthritis (RA) is an immune-mediated disorder caused by loss of immunological self-tolerance which generates systemic and chronic inflammation of synovial tissues that finally leads to cartilage and bone destruction. Extraarticular comorbidities related to vascular, metabolic and nervous systems are also exhibited in RA patients. Multiple genes mainly related to immune system, epigenetic changes, microRNA-expression patterns and environmental stimuli (tobacco, infections, diet, etc.) are involved in the pathogenesis of RA disease resulting in chronic inflammation of the synovium characterized by innate and adaptive cell infiltrates in the joints (1, 2).

The prevalence of RA is 0.24% worldwide and is more common in women than in men (ranging 1:2 to 1:3 ratios men/women). In addition, RA patients have a higher risk of mortality than the general population which is mostly caused by cardiovascular diseases, respiratory diseases and infections (3). Current therapies for the management of RA mainly target the immune system and involve non-steroidal anti-inflammatory drugs (NSAIDs), corticosteroids and synthetic and biological disease-modifying antirheumatic drugs (DMARDs). Despite this variety of drugs, RA is a lifelong disease without definitive cure for most patients. Furthermore, 10-15% of RA patients are refractory or intolerant to existing treatments (4, 5). New therapeutic approaches are needed and, in this sense, mesenchymal stem/stromal cells (MSCs) have emerged as a new alternative treatment for RA patients thanks to their well-documented immunomodulatory and regenerative properties (6, 7).

Numerous immune responses and mechanisms of action have been described for the immunomodulatory effect of MSCs in both preclinical and clinical studies of RA, although thus far the precise mechanism of action remains to be fully defined (8, 9). Different animal models of RA have been used to screen the mechanisms involved in MSC-based therapy. Among these, the majority of preclinical studies have been conducted in collagen-induced arthritis (CIA) mouse model (9) as it is widely considered the experimental animal model of arthritis that best resembles systemic immune responses of human RA (10, 11). In the CIA model, arthritis is induced by one or multiple injections of type II collagen (CII; the major constituent collagen form of articular cartilage) one, two or three weeks apart which activates both CII-reactive T (mostly Th17) and B cell responses (12). However, among the drawbacks of the CIA model are the low incidence of the disease in numerous mouse strains commonly used in immunological studies, a prolonged induction phase of at least 4 to 6 weeks, together with the low number of mouse strains that are susceptible to developing arthritis. These limitations have led to the development of alternative experimental models of RA (9). In this line, K/BxN serum transfer-induced arthritis (STIA) model (13) has been developed as a very useful *in vivo* model that mimics many human RA disease characteristics (14). To induce K/BxN STIA, serum from arthritic transgenic K/BxN mice is transferred to naïve mice and manifestations of arthritis occur a few days later with nearly a 100% incidence in many mouse strains. The inflammatory response is driven by autoantibodies against self-antigen glucose-6-phosphate isomerase (G6PI), which involves different immune mediators such as cytokines, chemokines, complement factors, integrins and toll-like and Fc receptors. These autoantibodies form immunocomplexes (ICs) that induce the activation of innate immune cells such as neutrophils, macrophages and mast cells. More recently, distinct fibroblast and macrophage subsets located in the synovial tissue have been identified (15–18). Interestingly, many features of the K/BxN STIA model mimic key mechanisms of the innate immune cell-driven effector phase of RA disease (14).

In this study, we aimed to evaluate the immunomodulatory potential of allogeneic adipose-derived MSC-based therapy in a K/BxN serum transfer-induced arthritis model. Unexpectedly, our data suggest that allogeneic adipose-derived MSC-based therapy was unable to modulate the course of the disease in arthritic mice despite the various experimental MSC-infusion protocols and parameters tested.

## Materials and methods

### Generation and characterization of murine adipose-derived mesenchymal stem/stromal cells

To generate murine adipose-derived mesenchymal stem/stromal cells, adipose tissue from female BALB/cJ mice was cut and digested with collagenase A at a final concentration of 2 mg/ml for 2-4 hours at 37°C. Digested samples were filtered and centrifuged. Cell pellets were seeded and cultured in MesenCult medium for mouse MSCs and 1% penicillin/streptomycin. For *in vitro* and *in vivo* studies, MSCs were used at passages 4 to 8.

MSCs were immunophenotypical and functional characterized as was previously described (19). For phenotypic analysis, the following monoclonal antibodies were used: CD45, CD3, CD45R/B220, CD34, Gr1, CD29, CD73 and CD90 based on the minimal criteria defined by the International Society for Cellular Therapy (ISCT) (20). For the immunosuppression assay, carboxyfluorescein diacetate N-succinimidyl ester (CFSE) labelling was used. Splenocytes were resuspended in 10 µM CFSE and incubated under shaking conditions at 37°C for 10 min. CFSE-labelled splenocytes were activated with 12.5 µg/ml αCD3/αCD28 and 180 U/ml IL-2. Ratio of 1:200 MSCs:splenocytes was used. At day 3, cells were harvested and labelled with αCD4 antibodies. Cell proliferation of the CFSE labeled-CD4^+^ DAPI^-^ (4’, 6-Diamidino-2-Phenylindole, Dihydrochloride) population (viable lymphocytes) was determined by flow cytometry (**Supplementary Figure 1**).

### K/BxN serum transfer-induced arthritis experimental design

K/BxN mice were generated by crossing B6.KRN TCR transgenic mice and non-obese diabetic (NOD) mice. Mice were maintained in the specific pathogen-free (SPF) facility of in the Center for Experimental Biomedicine (CEBEGA) from the University of Santiago de Compostela. Animal care was in compliance with Spanish regulations on the protection of animals used for experimental and other scientific purposes (Real Decreto 1386/2018). The experimental protocols were approved by the animal care and use Committee of the CIEMAT and Comunidad de Madrid (PROEX 241.4/20).

Serum was collected from 4- to 8-week-old arthritic K/BxN mice, pooled and stored at –80°C until use. Arthritis was induced by transfer of one or two doses of 100 μl of K/BxN serum two days apart into 9- to 12-week-old male C57BL/6J mice from the Jackson Laboratory or DBA1/J and C57BL/6NRj-Rag2tm1Ciphe/Rj mice from Janvier Labs by intraperitoneal (IP) or intravenous (IV) injections with or without an additional intraperitoneal infusion of 100 μg of lipopolysaccharide (LPS) three days later. One or two doses of MSCs were intravenously (0.5 × 10^6^ cells/mouse) or intraperitoneally (3 × 10^6^ cells/mouse) infused on the indicated day. Arthritis severity was analyzed by the sum of thickness (mm) of each four limbs measured by a calliper and total body weight.

### Hematologic and flow cytometry analyses

Peripheral blood samples were examined by an automated blood cell-counter (hematology analyzer, Sysmex). Peripheral blood (100 µl) was also surface-stained with the following antibodies: CD3, CD4, CD11b, Ly6G and Ly6C. Cells were collected on a BD LSR Fortessa flow cytometer. Data were analyzed using FlowJo software.

### Statistical analysis

Normal distribution was analyzed by the Shapiro-Wilks test. The parametric student T test was used for normal distribution and non-parametric Mann-Whitney U test was used for non-normal distribution. Analysis was performed using the GraphPad Prism 9.2.0.

## Results

### Assessment of a therapeutic protocol

**Figure 1:**
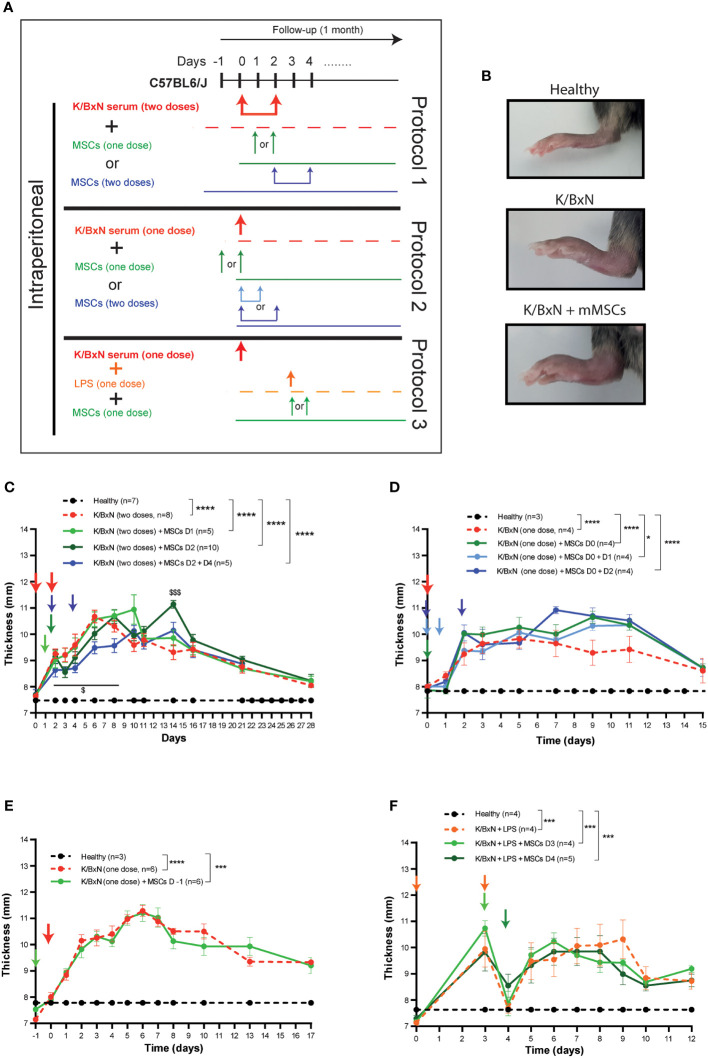
Experimental design of K/BxN serum-transfer induced arthritis and treatment with allogeneic adipose-derived MSCs. **(A)** Experimental design using two doses (Protocol 1) or a single dose (Protocol 2) of IP infusion of K/BxN sera infusion. Protocol 3 shows LPS treatment at day 3, with one or two doses of IP infusion of allogeneic adipose-derived MSCs. **(B)** Representative pictures of hind paws at day 6 following two doses of serum from K/BxN arthritic mice and two doses of MSCs at days 2 and 4. Systemic joint inflammation following two doses of serum from K/BxN mice without or with a single dose of IP infusion of MSCs at day 1 or day 2 or two doses at days 2 and 4 **(C)**. Systemic joint inflammation following one dose of serum from K/BxN mice and a single dose of IP infusion of MSCs at day 0 or two doses at days 0 and 1 or at day 0 and 2 **(D)** or 24 hours before the K/BxN serum infusion **(E)**. Systemic joint inflammation following one dose of serum from K/BxN arthritic mice with a single dose of IP infusion of LPS at day 3 following by a single dose of MSCs intraperitoneally at day 3 or day 4 **(F)**. Data are presented as mean and standard error of the mean of systemic joint inflammation calculated by the sum of thickness (mm) of the four paws measured by a calliper. Significance of the cumulative thickness was analyzed by the Mann-Whitney U test represented by *p < 0.05, *** p < 0.001 and ****p < 0.0001, any group of mice vs heathy mice and $ p < 0.05 and $$$ p < 0.001 K/BxN + MSCs at day 2 mice vs K/BxN mice. n, number of mice.

According to the most commonly used protocol, two intraperitoneal (IP) infusions of serum derived from K/BxN arthritic mice were infused at day 0 and day 2 into C57BL/6J mice (Protocol 1, **Figure 1A**). Mice developed systemic joint inflammation with an incidence of 100% compared to healthy mice from 24 hours and over a period of three weeks, which was measured by the sum of every paw thickness with a calliper (**Figure 1C**). The peak of join inflammation was reached by day 6 from the first K/BxN serum infusion with a significant total thickness of 10.7 ± 0.2 mm in arthritic mice compared to 7.5 ± 0.4 mm in healthy mice. This increase in the joint inflammation was paralleled by a 10% greater decrease in body weight over two weeks than that seen in healthy mice (**Supplementary 2A**). A single IP infusion of MSCs following K/BxN serum-transfer was performed during the onset of the joint inflammation at day 1 or at day 2. A slight decrease in joint inflammation was observed at day 3 (8.6 ± 0.2 mm) compared to untreated arthritic mice (9.2 ± 0.3 mm, **Figure 1C**), however these differences were not significant. Total body weight remained unchanged upon a single IP infusion of MSCs (**Supplementary Figure 2A**). To enhance the mild immunomodulatory effect observed, two doses of MSCs were infused at days 2 and 4 (**Figure 1C** **).** In this instance, the decrease in systemic joint inflammation was maintained from day 2 to day 8 in MSC-treated K/BxN mice compared to untreated arthritic mice. No significant differences were noticed in systemic joint inflammation (**Figure 1C**). Strikingly, an increase in systemic joint inflammation was observed by day 7 following MSC infusion compared to untreated arthritic mice, which was significantly higher when MSC infusion was performed at day 2 (**Figure 1C**). These results were also confirmed macroscopically as shown in **Figure 1B**. We then decided to simplify the induction phase of the inflammation protocol by administering a single IP infusion of serum derived from K/BxN arthritic mice into C57BL/6J mice (Protocol 2, **Figure 1A**). A single dose of K/BxN serum induced milder systemic joint inflammation compared to the standard K/BxN STIA protocol as shown by the lower systemic joint inflammation at day 5 (9.8 ± 0.4 mm) than with two IP infusions of K/BxN serum (10.7 ± 0.2 mm). In this instance, a similar incidence was achieved (100%) compared to healthy mice from 24 hours for two weeks instead of the three weeks observed following two IP infusions of K/BxN serum (**Figure 1D**). The reduced increase in systemic joint inflammation was paralleled by a lower decrease of body weight over one week compared to healthy mice which received two intraperitoneal infusions of K/BxN serum (**Supplementary 2A, B**). A single IP infusion of MSCs the same day as the K/BxN serum-transfer produced a mild but not significant decrease in systemic joint inflammation for one day (**Figure 1D**). Total body weights remained unaltered (**Supplementary Figure 2B**). Aiming to sustain the immunomodulatory effect, an additional intraperitoneal infusion of MSCs was assessed at day 1 or day 2. No clear immunomodulatory effects in systemic joint inflammation were detected (**Figure 1D**). On the contrary, a mild increase in systemic joint inflammation was observed following IP MSC infusion in MSC-treated arthritic mice compared to untreated arthritic mice (**Figure 1D**). No effects on total body weights were observed (**Supplementary Figure 2B**).

### Assessment of a prophylactic protocol

Since no beneficial effects were observed following infusion of MSCs during the onset of joint inflammation, we decided to infuse MSCs intraperitoneally prophylactically 24 hours before K/BxN serum infusion. A similar degree of joint inflammation based on paw thickness and total body weights were observed in the MSC-treated and untreated arthritic mice (**Figure 1E** and **Supplementary Figure 2C**).

### Assessment of a LPS-induced boosting of arthritis protocol

The K/BxN STIA model shares many similarities with the collagen antibody-induced arthritis (CAIA) model (21), where systemic joint inflammation is driven by anti-collagen antibodies. In the CAIA model, two studies have demonstrated the immunomodulation of MSC-based therapy (22, 23). In the CAIA model, joint inflammation is triggered upon infusion of lipopolysaccharide (LPS) three days after collagen-against antibody is infused. Hence, we decided to treat the mice with LPS intraperitoneally three days after infusion of serum from K/BxN arthritic mice into C57BL/6J mice (**Figure 1A**, Protocol 3). Similar levels of systemic joint inflammation (**Figure 1F**) and body weight losses (**Supplementary Figure 2D**) were observed in comparison to a single intraperitoneal dose of K/BxN serum without LPS infusion and to healthy mice (**Figure 1D**, **Supplementary Figures 2B, C**). In contrast to previously assessed protocols, 24 hours after IP infusion of LPS, a rapid and transitory reduction in systemic joint inflammation was noticed (**Figure 1F**). By day 5, the degree of systemic joint inflammation was similar to day 3 and remained significantly increased compared to control mice for at least one week. In any case, the immunomodulatory effects were not observed following a single dose of intraperitoneal infusion of MSCs at day 3 or at day 4 (**Figure 1F** and **Supplementary Figure 2D**) in contrast to what was previously reported in the CAIA model (22, 23).

### Evaluation of different routes of administration for the MSCs and K/BxN serum

**Figure 2:**
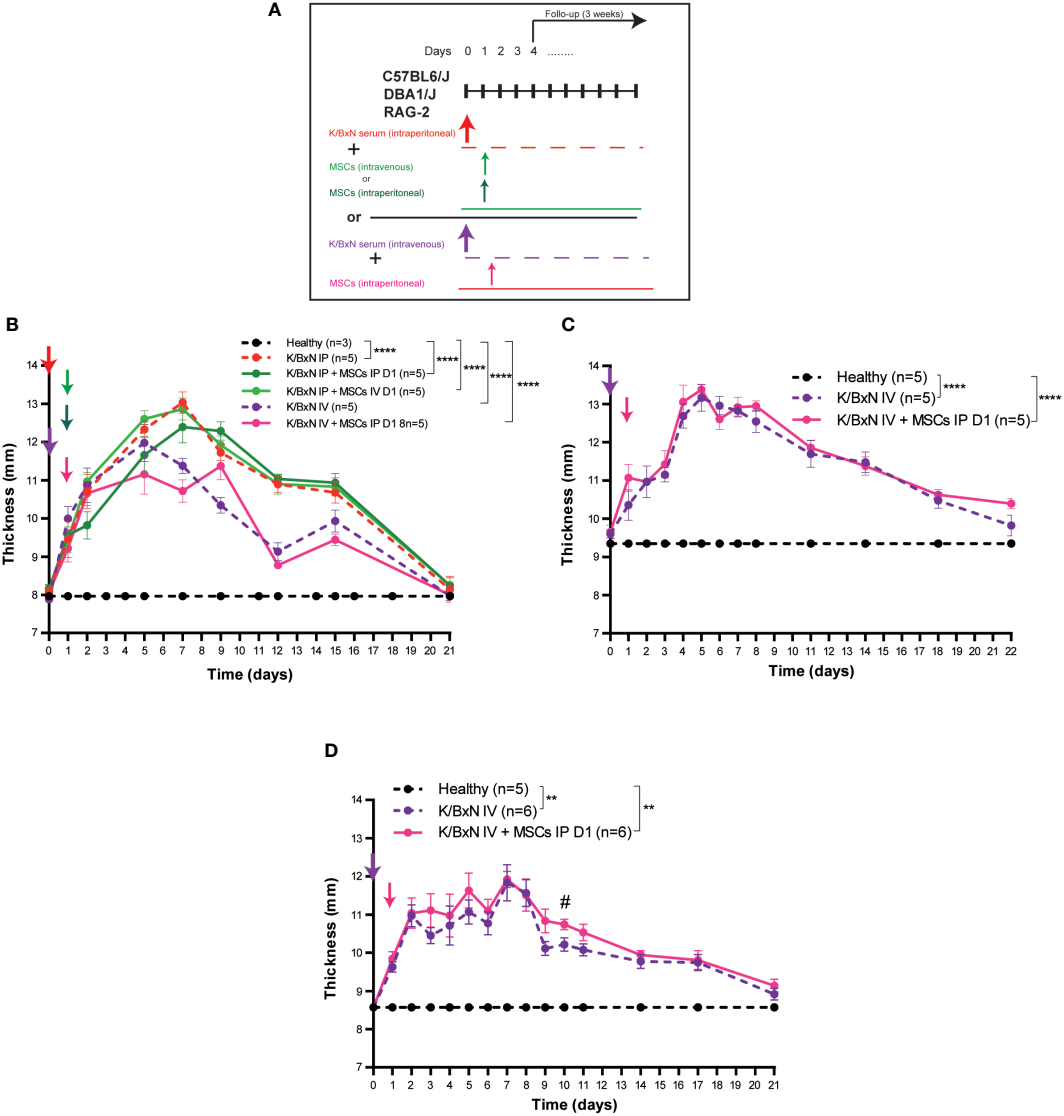
Experimental design and arthritis status of a single dose of IP versus IV infusion of K/BxN serum-transfer in C57BL/6J, DBA/J or RAG-2 mice and treatment with allogeneic adipose-derived MSCs using a different route of administration. Experimental design **(A)** and systemic joint inflammation following a single IP or IV serum infusion from K/BxN arthritic mice and a single dose (at day 1) of IV (0.5 x 10^6^ MSC/mouse) or IP (3 x 10^6^ MSC/mouse) infusion of MSCs in C57BL/6J **(B)**, DBA1/J **(C)** or RAG-2-deficient mice **(D)**. Data are presented as mean and standard error of the mean of systemic joint inflammation calculated by the sum of thickness (mm) of the four paws measured by a calliper. Significance of the cumulative thickness was analyzed by the Mann-Whitney U test represented by **p < 0.01 and ****p < 0.0001, any group of mice vs heathy mice, ^#^ p < 0.05 K/BxN + MSCs vs K/BxN mice. n, number of mice.

We reasoned that potential interaction between the K/BxN serum and MSCs in the peritoneal cavity may occur causing a blockade of the biological function of MSCs. Therefore, different routes of administration for the K/BxN serum and the MSCs were assayed (**Figure 2A**). A single IV infusion of serum from K/BxN arthritic mice into C57BL/6J mice induced systemic joint inflammation with the maximum level at day 5 (12.0 ± 0.5 mm) **Figure 2B**). No immunomodulatory effect was observed following intraperitoneal infusion of MSCs 24 hours after the IV infusion of serum from K/BxN mice similar to what was observed when both K/BxN serum and MSC were intraperitoneally infused (**Figures 2B**, **1D**, respectively). No effects on total body weights were noticed (**Supplementary Figure 2E**). In neither of the two protocols tested there was a significant immunomodulatory effect based on systemic joint inflammation and total body weights were observed following MSC-based therapy. These results ruled out any potential inhibitory effect due to administration of K/BxN and MSCs through the IP route of administration.

### Assessment of mouse strain susceptibility for MSC-based therapy

The DBA1/J strain of mice is more susceptible to arthritis development than C57BL/6J mice (24). As we and others have previously demonstrated, MSC-based therapy delayed the progression of established collagen-induced arthritis (CIA) (9, 25, 26) and collagen antibody-induced arthritis (CAIA) (22, 23). In both instances, the DBA1/J strain of mice was used. Aiming to know the potential impact of the major histocompatibility complex (MHC) context on the immunomodulatory effect of MSC-based therapy in the K/BxN STIA model, we carried out the experimental design depicted in **Figure 2A** using the DBA1/J mice to avoid the potential interaction between the K/BxN serum and MSCs in the peritoneal cavity and the limitation of MSC dose by IV route of administration. In this protocol, serum from K/BxN arthritic mice was infused intravenously. A higher level of systemic joint inflammation at day 5 (13.2 ± 0.4 mm, **Figure 2C**) together with increased body weight losses (**Supplementary Figure 2F**) were observed in DBA1/J mice compared to C57BL/6J mice (12.0 ± 0.5 mm, **Figure 2B** and **Supplementary Figure 2E**). No immunomodulatory effects upon MSC infusion at day 1 in terms of systemic joint inflammation (**Figure 2C**) and total body weight losses (**Supplementary Figure 2F**) were observed, which suggests that the lack of immunomodulatory effect of MSC-based therapy was not due to intrinsic mouse strain susceptibility to immunomodulation mediated by MSC-based therapy.

### Evaluation of immune context

According to previous studies, the myeloid compartment is the first target for MSC-based therapy (26) and innate immune-mediated responses are the key orchestrators of systemic joint inflammation in the K/BxN STIA model (14). Based on these observations, K/BxN serum was intravenously infused into Rag-2-deficient mice in the absence of adaptive B and T cell responses. As shown in **Figure 2D**, systemic joint inflammation reached similar levels in RAG-2-deficient C57BL/6J mice (11.8 ± 0.5mm, **Figure 2D**) and immunocompetent C57BL/6J mice (12.0 ± 0.5 mm, **Figure 2B**), confirming that adaptive B and T cell responses are not required to induce systemic joint inflammation in the K/BxN STIA model. Total body weights were similar to C57BL/6J mice (**Supplementary Figures 2E, G**, respectively). No immunomodulatory effects caused by MSC-based therapy were observed in terms of systemic joint inflammation (**Figure 2D**) or total body weight (**Supplementary Figure 2G**).

### Phenotypic analysis of leukocyte populations in peripheral blood following cell therapy with MSCs in K/BxN serum-transfer induced arthritis model

Some of the immune responses involved in the therapeutic effect of MSC-based therapy in collagen-induced arthritis and collagen-antibody induced arthritis models have been previously described (9). The different immune mechanisms involved in the development of systemic joint inflammation induction and/or development in these experimental arthritis models could be the reason for the lack of therapeutic effect by MSC-based cell therapy observed in the K/BxN STIA model compared to CIA and CAIA models of arthritis. To further investigate these differences, we carried out hematological and phenotypical analyses of peripheral blood populations in the K/BxN STIA model following the infusion of MSCs.

**Figure 3:**
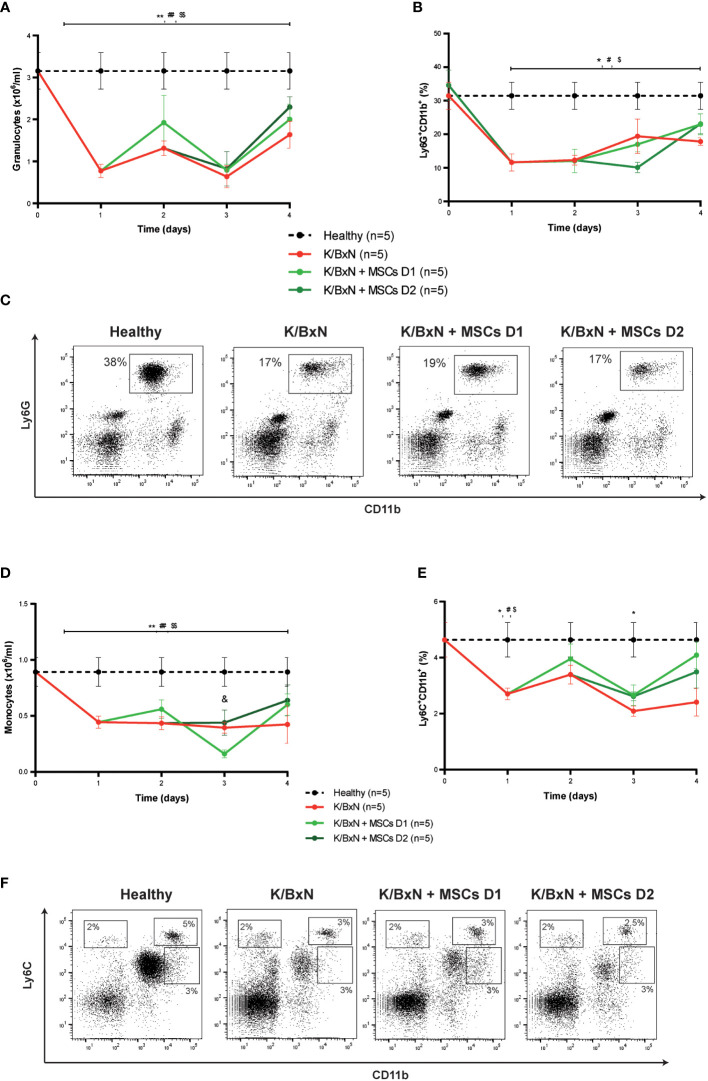
Hematological and flow cytometry analyses of myeloid cells in healthy and K/BxN serum transfer-induced arthritic mice treated and non-treated with MSCs. **(A)** Number of granulocytes (x10^6^/ml) and **(B)** frequency of Ly6G^+^CD11b^+^ myeloid cells (%) in peripheral blood 4 days after from the first transfer of serum from K/BxN mice. **(C)** Representative dot-plots at day 3 of Ly6G^+^CD11b^+^ myeloid cells. **(D)** Number of monocytes (x10^6^/ml), **(E)** frequency of Ly6C^+^CD11b^+^ myeloid cells (%) in peripheral blood 4 days after from the first K/BxN serum-transfer. **(F)** Representative dot-plots at day 3 of different Ly6C-expressing myeloid populations. Number of granulocytes and monocytes were obtained by an automated blood cell-counter and frequency of Ly6G-expressing and Ly6C-expressing CD11b^+^ myeloid cells were obtained by flow cytometry. Data are presented as mean and standard error of the mean. Significance of the cumulative number or frequency of the different populations was analyzed by the Mann-Whitney U test represented by *p < 0.05 and **p < 0.01 K/BxN vs heathy; ^#^ p < 0.05 and ^##^ p < 0.01 K/BxN + mMSCs day 1 vs healthy, ^$^ p < 0.05 and ^$$^ p < 0.01 K/BxN + mMSCs day 2 vs healthy and ^&^ p < 0.05 K/BxN + mMSCs day 1 vs K/BxN mice. n, number of mice.

Total white blood cells in peripheral blood decreased compared to healthy mice (data not shown), mainly due to a significant decrease of granulocytes (1.1 ± 0.2 x10^6^/ml, **Figure 3A**) and to a lesser extent of monocytes (0.42 ± 0.01 x10^6^/ml, **Figure 3D**) compared to healthy mice (3.2 ± 0.4 x10^6^/ml and 0.9 ± 0.1x10^6^/ml, respectively, **Figures 3A, D**) measured by a hematological counter. By flow cytometry, a decrease in the percentage of Ly6G^+^CD11b^+^ neutrophils (19.2 ± 1.7%, **Figures 3B, C**) and to a lesser extent in Ly6C^+^CD11b^+^ monocytes (3.6 ± 0.5%, **Figures 3E, F**) was observed in peripheral blood of K/BxN STIA mice compared to healthy mice (36.2 ± 4.4% and 5.8 ± 2.8%, respectively). No differences were observed in other populations of monocytes such as Ly6C^+^CD11b^int^ and Ly6C^int^CD11b^+^ cells (**Figure 3F**). No clear effects on neutrophils (**Figure 3A**) or Ly6G^+^CD11b^+^ myeloid populations (**Figures 3B, C**) and on monocytes (**Figure 3D**) or Ly6C^+^CD11b^+^, Ly6C^+^CD11b^int^ and Ly6C^int^CD11b populations (**Figures 3E, F**) in the peripheral blood were observed following MSC infusions at day 1 or at day 2.

Considering adaptive immune responses, a slight increase, but not significantly, in CD4^+^CD3^+^ and CD8^+^CD3^+^ T cells was observed after the K/BxN serum transfer compared to healthy mice. No clear effects were observed following MSC-based therapy in any of other T cell populations studied (**Supplementary Figure 3**).

These results demonstrate that MSC-based therapy fails to provide any immunomodulatory effect on systemic joint inflammation in the K/BxN STIA model despite the number of different experimental parameters tested.

## Discussion

In previous studies, we demonstrated that MSC-based therapy is able to modulate systemic inflammation in the collagen-induced arthritis (CIA) model (25–27). Despite these promising results, the low incidence, high variability, MHC restriction and large period of induction of systemic inflammation in the CIA model have encouraged us the search for an alternative experimental arthritis model for MSC-based therapy studies. In this sense, K/BxN serum-transfer arthritis (STIA) has emerged as an alternative arthritis model for MSC-based therapy studies. This model develops systemic joint inflammation very rapidly upon induction of arthritis with a nearly 100% incidence in most of mouse strains assessed. Most importantly, many of the mechanisms of action participating in the inflammatory responses mimic numerous features of human RA disease (14). Additionally, in contrast to the CIA model in which adaptive immune system plays the major role in the induction phase of joint inflammation (12), in the K/BxN STIA model, mainly innate immune cells are implicated in the induction phase of the joint inflammation (14) as shown by the development of K/BxN STIA in RAG-deficient mice (28–30). Since we and others have previously demonstrated that the myeloid compartment is the first target in MSC-based therapy in RA as well as in experimental colitis (19, 26), the K/BxN STIA model could be conceived of as an optimal experimental arthritic model to study MSC-based therapy.

Preliminary experiments were set up to define the optimal protocol for MSC-based therapy in the K/BxN STIA model based on the pioneering studies developed by Monach and collaborators (31). Thus, we infused two intraperitoneal infusions of serum derived from K/BxN arthritic mice into C57BL/6J mice. Systemic joint inflammation started rapidly, 24 hours after the first K/BxN serum infusion, with an incidence of 100% in C57BL/6J mice as described (14). These results confirm that the K/BxN STIA model could be an alternative arthritic model in C57BL/6J MHC context (H-2b) in which most studies are conducted. Furthermore, C57BL/6J mice are naturally refractory to developing CIA in contrast to DBA/1 mice (H-2q) (32).

Based on numerous studies in experimental immune-mediated disorders, better efficacy is achieved when the MSCs were infused during the early phases of the disease (9, 33). We therefore infused 3x10^6^ MSCs per mouse during the onset of joint inflammation soon after serum administration from K/BxN arthritic mice. The dose used in these experiments was chosen based on the most commonly used MSC dosage with therapeutic efficacy in preclinical studies of arthritis (range from 1x10^6^ to 10x10^6^ MSCs per mouse) (9). A slight and transient immunomodulatory effect on systemic joint inflammation was observed following MSC infusion although a few days later systemic joint inflammation increased in MSC-treated arthritic mice compared to untreated arthritic mice.

To avoid a potential deleterious effect in the beneficial effects of MSCs by components present in the sera derived from K/BxN arthritic mice within the peritoneal cavity, K/BxN sera were infused intravenously. It is widely described that although MSC biodistribution depends on the route of administration, their therapeutic effect is systemic in different immune-mediated disorders (9, 33). In this scenario, we also observed a mild and transient decrease in systemic joint inflammation following intraperitoneal MSC infusion followed by a systemic joint inflammation increase. The mild and transient effect observed after MSC infusion could be explained by a rapid trafficking of leukocytes from the inflamed joints to the peritoneal cavity where MSCs were administered as has previously been described (22, 34).This transient immunomodulatory effect did not occur when MSCs were intravenously infused following intraperitoneal infusion of serum from K/BxN mice, most likely due to the murine MSC cell dose used (0.5x10^6^ MSCs per mouse) aiming to avoid mouse survival. This observation suggests that the lack of therapeutic effect by MSCs in the K/BxN STIA model was not caused by K/BxN serum administered within the peritoneal cavity.

Based on these results and since it is widely described that MHC context has a great impact on the development of systemic joint inflammation depending on the particular challenge used for arthritis induction, we performed the K/BxN STIA protocol in DBA1/J mice. DBA1/J mice were previously used in our studies in MSC-based therapy in CIA (25–27) and in CAIA (22, 23) models where successful results in terms of therapeutic efficacy have been reported. As expected, DBA1/J mice were more susceptible to developing systemic joint inflammation after K/BxN serum infusion compared to C57BL/6J mice thus confirming their higher susceptibility to arthritis. However, in contrast to what was observed in MSC-based therapy following collagen or antibody against collagen challenges for arthritis induction in the DBA1/J strain of mice, no immunomodulatory effects were observed following MSC infusion and G6PI-against antibody challenge. In the MHC context, it should be pointed out that allogeneic and xenogeneic MSCs have shown similar therapeutic effects in different arthritic models (9).

In the K/BxN STIA model, ICs are formed systemically since G6PI is ubiquitously present. These ICs activate inflammatory immune cells which subsequently release mediators that increase vascular permeability facilitating access of ICs and antibodies to the synovial tissue. In the CAIA model in which MSC-based therapy has a therapeutic effect, the collagen II antigen is mainly present in the joint and ICs cannot be formed systemically, therefore, an additional trigger is needed to ensure that antibodies against CII can access the joints. In this instance, an intraperitoneal infusion of lipopolysaccharide (LPS) is necessary to increase the disease incidence and severity of arthritis (21, 35). LPS itself also activates joint macrophages and fibroblasts by activating the TLR-4/NFκB pathway (36). Moreover, LPS infusion is used to increase disease incidence and severity in strains of mice that are less susceptible to arthritis induction by collagen challenge (36). Based on these results, we infused LPS three days following serum infusion to mimic the CAIA model protocol in which MSC-based therapy modulates systemic joint inflammation (22, 23). Strikingly, LPS infusion provoked a significant, although transient, decrease in systemic joint inflammation likely because of the migration of leukocytes to the peritoneal cavity where the LPS was infused. In this scenario, no therapeutic effects of MSC-based therapy were observed suggesting that the increase in vascular permeability and activation of joint macrophages are not involved in the beneficial effects of MSC-based therapy.

Our previous results in T and B cell-deficient colitic Rag-1 mice pointed out the innate system as the main target for MSC-based therapy even though adaptive immune responses can hinder some of the MSC therapeutic effects in immunocompetent mice (19). Based on these results, we conducted the K/BxN STIA model in RAG-2-deficient C57BL/6J mice aiming to avoid the potential masking effect of the inflammatory adaptive immune system in the therapeutic effect of MSCs. In the absence of T and B cells, the level of systemic joint inflammation was similar to fully immunocompetent C57BL/6J mice confirming that the adaptive immune system does not contribute to the induction/development of systemic joint inflammation following K/BxN serum infusion. In this scenario, no immunomodulatory effects of MSCs were observed, either.

All of these results point out that mesenchymal stem/stromal cell-based therapy fails to provide any beneficial effect in the K/BxN serum-transfer induced arthritis model in any of the scenarios tested in this study. These results suggest that the immune responses targeted by MSCs are not involved in the induction phase and/or development of systemic joint inflammation in the K/BxN serum transfer-induced arthritis model.

Analysis of peripheral blood revealed a significant decrease in neutrophil (Ly6G^+^CD11b^+^) and monocyte (Ly6C^+^CD11b^+^) populations soon after K/BxN serum infusion, likely due to their migration to peripheral tissues, in contrast to what was observed in the CIA model in which the collagen II boost injection produced high numbers of neutrophils and monocytes in the peripheral blood (26, 37). These high levels of myeloid cells are most likely mobilized from the bone marrow and spleen (37, 38). Moreover, in the CAIA model, LPS infusion increases the levels of myeloid cells in peripheral blood (39). Reduced numbers of neutrophils and monocytes in the peripheral blood of K/BxN STIA mice suggest that these populations migrate from the periphery to the joints soon after K/BxN serum infusion without mobilizing them from their natural reservoirs. This suggests that this process could be an important event required for the beneficial effects of MSC-based therapy.

It has been described that systemic joint inflammation produced by K/BxN serum transfer could be modulated by neutrophil depletion, neutrophil migration or neutrophil differentiation blockage (40, 41) since the majority of the leukocytes found in the joints are neutrophils (14, 29). However, no effects on levels of neutrophils in peripheral blood were observed upon MSC infusion which could explain why MSC-based therapy failed to modulate systemic joint inflammation in this arthritic model. Despite this hypothesis, in previous studies we observed that MSC-based therapy was able to modulate systemic joint inflammation in the CIA model without altering the increased neutrophil levels in peripheral blood (26). Strategies to target neutrophils also modulated systemic joint inflammation in CIA (42) and CAIA (43, 44) models. All of these observations and previous studies with MSC-based therapy in arthritic models point out that the neutrophil population is not the main target for MSC-based therapy in experimental arthritis.

On the other hand, no significant effects were observed on the monocytic (Ly6C^+^CD11b^+^ cells) populations in peripheral blood following MSC-based therapy. In contrast to these data, in our previous studies of the CIA model, we observed that the therapeutic effects mediated by MSCs were accompanied by a transient increase of monocytes (Ly6C^+^CD11b^+^) in peripheral blood that paralleled an increase of regulatory macrophages in the lymph nodes (26). This points out this population as the main cells responsible for MSC therapeutic effect according to other studies (22, 45–49). Fibroblast, macrophage and osteoclast subsets are the main orchestrators of the inflammation in the joint synovium. Destruction of articular bone has been demonstrated to be almost exclusively mediated by osteoclasts that are replaced by a perpetual supply of osteoclast precursors (50) and by monocytic/macrophage cells although the precise mechanisms involved are unknown (16, 17, 51). Though the origin and role of synovial macrophages in inflammatory joints remain unclear, it is assumed that synovial macrophages expressing CX_3_CR1 are differentiated from tissue-resident macrophages (16) and from circulating monocytes. Blood monocytes display different phenotypes: “classical” CCR2-expressing Ly6C^high^ monocytes and “nonclassical” CX_3_CR1-expressing Ly6C^low^ monocytes. Some studies claimed that only “nonclassical” monocytes are the ones implicated in the initiation and the progression of arthritis in K/BxN STIA mice as a consequence of their development into synovial macrophages (17, 51, 52). In fact, in K/BxN STIA, although systemic clodronate-mediated depletion of macrophages resulted in a drastic amelioration of the arthritis (53, 54), CCR2 deficiency (54) did not alter arthritis development whereas CX_3_CR1 deficiency produced exacerbated inflammation (16, 17). In contrast to these results, in CIA mice as well as in CAIA models, CCR2 deficiency (55, 56) or CCR2 pathway blockage (43, 57, 58) is associated with an increase in clinical signs and joint damage whereas in CIA mice, CX_3_CR1 deficiency is protective (59). In CIA, tissue-resident macrophages gradually decrease from initial developmental stage (60) and CCR2-expressing Ly6C^high^ monocytes differentiate into CX_3_CR1-expressing Ly6C^low^ in the synovium microenvironment and express macrophage markers that finally induce osteoclastogenesis (61). The CCR2-expressing Ly6C^high^ monocyte population is enlarged in bone marrow and spleen by inflammation-induced hematopoiesis. Furthermore, it is highly migratory toward the CCL2 chemokine increased in the joints during the arthritis development. In contrast to CCR2-expressing Ly6C^high^ monocyte population, CX_3_CR1-expressing Ly6C^low^ monocytes is downregulated in bone marrow in collagen-induced arthritis mice (37). All of these data suggest that different monocyte populations are implicated in the induction/development of systemic joint inflammation among the arthritic models although more mechanistic studies are needed to determine possible functional roles of classical and nonclassical monocytes in the development of synovial macrophages and osteoclasts in the different models of arthritis. In this line, recent insights into the heterogeneity of tissue-resident synovial cells, including macrophages and fibroblasts, during the different stages of the human RA disease have revealed the importance of these populations as targets for the success of treatments for RA (62). At present, a significant number of preclinical studies in different RA models have shown that systemic administration of MSCs can reduce systemic joint inflammation (9) although these successful results in animal models of RA have not been replicated in phase I/II clinical trials (8) which can be explained by the heterogeneity in the status of the tissue-resident synovial cells in the RA patients enrolled, most of them with a long history of the disease.

A recent study demonstrated that semaphorin 3B (sema3B), a secreted protein, is implicated in ameliorating the migration and invasiveness of fibroblast-like synoviocytes (FLS) in RA. Both in K/BxN STIA and in RA patients, delayed progression of joint damage upon administration of recombinant sema3B was observed. These data clearly suggest that K/BxN STIA can be effectively modulated in preclinical studies once the target mechanism has been identified (63).

## Conclusion

K/BxN STIA model is not adequate for preclinical studies of MSC-based therapy since no immunomodulatory effects were observed following mesenchymal stem/stromal cell infusion. The potential reasons for the discrepancies in the efficacy of MSC-based therapy among the arthritic models are based on the differences in the pathogenesis of inflammation according to the different immune statuses and monocytic/macrophage balance in the inflamed mice among the different preclinical models of arthritis. These observations could help to identify those RA patients most likely to respond to MSC-based therapy that will clearly accelerate the clinical translation of MSC- based therapy for RA.

## Data availability statement

The original contributions presented in the study are included in the article/**Supplementary Material**, further inquiries can be directed to the corresponding author/s.

## Author contributions

Conceptualization: ML-S and MIG. Investigation and Methodology: ML-S, CC, AR-T and MIG. Investigation: ML-S, CC, A-T, and MIG. Formal Analysis and Validation: ML-S and MIG. Writing-Original Draft Preparation: ML-S and MIG. Writing-Review and Editing: ML-S, CC, AR-T and MIG. All authors contributed to the article and approved the submitted version.

## Funding

This work was supported by grants from “Instituto de Salud Carlos III (ISCIII)”, co-funded by the European Union (PIE15/00048, PI17/01161, PI21/01441, PI20/01266, RICORS; RD21/0017/00; funded by European Union-NextGenerationEU. Plan de Recuperación Transformación y siliencia) and Comunidad de Madrid (AvanCell, B2017/BMD-3692). CIBERER is an initiative of the “Instituto de Salud Carlos III” and the European Regional Development Fund (ERDF).

## Acknowledgments

The authors would like to thank Prof. Juan Antonio Bueren for the intellectual revision of the manuscript, Miguel A. Martin for the careful maintenance of mice and Norman A. Feltz for the revision of the language in the manuscript. All authors confirm that they had full access to all the data in the study.

## Conflict of interest

The authors declare that the research was conducted in the absence of any commercial or financial relationships that could be construed as a potential conflict of interest.

## Publisher’s note

All claims expressed in this article are solely those of the authors and do not necessarily represent those of their affiliated organizations, or those of the publisher, the editors and the reviewers. Any product that may be evaluated in this article, or claim that may be made by its manufacturer, is not guaranteed or endorsed by the publisher.
