# Corrigendum: Assessment of mesenchymal stem/stromal cell-based therapy in K/BxN serum transfer-induced arthritis

**DOI:** 10.3389/fimmu.2024.1384171

**Published:** 2024-05-08

**Authors:** Mercedes Lopez-Santalla, Carmen Conde, Angela Rodriguez-Trillo, Marina I. Garin

**Affiliations:** ^1^ Division of Hematopoietic Innovative Therapies, Biomedical Innovation Unit, Centro de Investigaciones Energéticas Medioambientales y Tecnológicas (CIEMAT), Madrid, Spain; ^2^ Centre for Biomedical Network Research on Rare Diseases (CIBER-ER) and Advanced Therapy Unit, Madrid, Spain; ^3^ Advanced Therapy Unit, Health Research Institute- Fundación Jiménez Díaz, University Hospital, Universidad Autónoma de Madrid (IIS-FJD, UAM), Madrid, Spain; ^4^ Laboratorio de Reumatología Experimental y Observacional, Instituto de Investigación Sanitaria de Santiago (IDIS), Hospital Clínico Universitario de Santiago de Compostela (CHUS), Servicio Gallego de Salud (SERGAS), Santiago de Compostela, Spain

**Keywords:** mesenchymal stem/stromal cell-based therapy, K/BxN serum transfer-induced arthritis, joint inflammation, cell therapy, immunomodulation

In the published article, there was a mistake in the Funding statement. The Funding statement for the “Instituto de Salud Carlos III (ISCIII)” was displayed as “Instituto de Salud Carlos III (ISCIII)” (PIE15/00048, PI17/01161, PI21/01441, PI20/01266, RICORS; RD21/0017/00’.

The correct statement is as follows:

‘This work was supported by grants from “Instituto de Salud Carlos III (ISCIII)”, co-funded by the European Union (PIE15/00048, PI17/01161, PI21/01441, PI20/01266, RICORS; RD21/0017/00; funded by European Union-NextGenerationEU. Plan de Recuperación Transformación y siliencia) and Comunidad de Madrid (AvanCell, B2017/BMD-3692). CIBERER is an initiative of the “Instituto de Salud Carlos III” and the European Regional Development Fund (ERDF)’.

The authors apologize for this error and state that this does not change the scientific conclusions of the article in any way. The original article has been updated.

