# Supplementary figures and images for: Assessment of mesenchymal stem/stromal cell-based therapy in K/BxN serum transfer-induced arthritis
Source: Front Immunol. 2022 Oct 10;13:943293. doi: 10.3389/fimmu.2022.943293 (PMC9589432; doi:10.3389/fimmu.2022.943293)

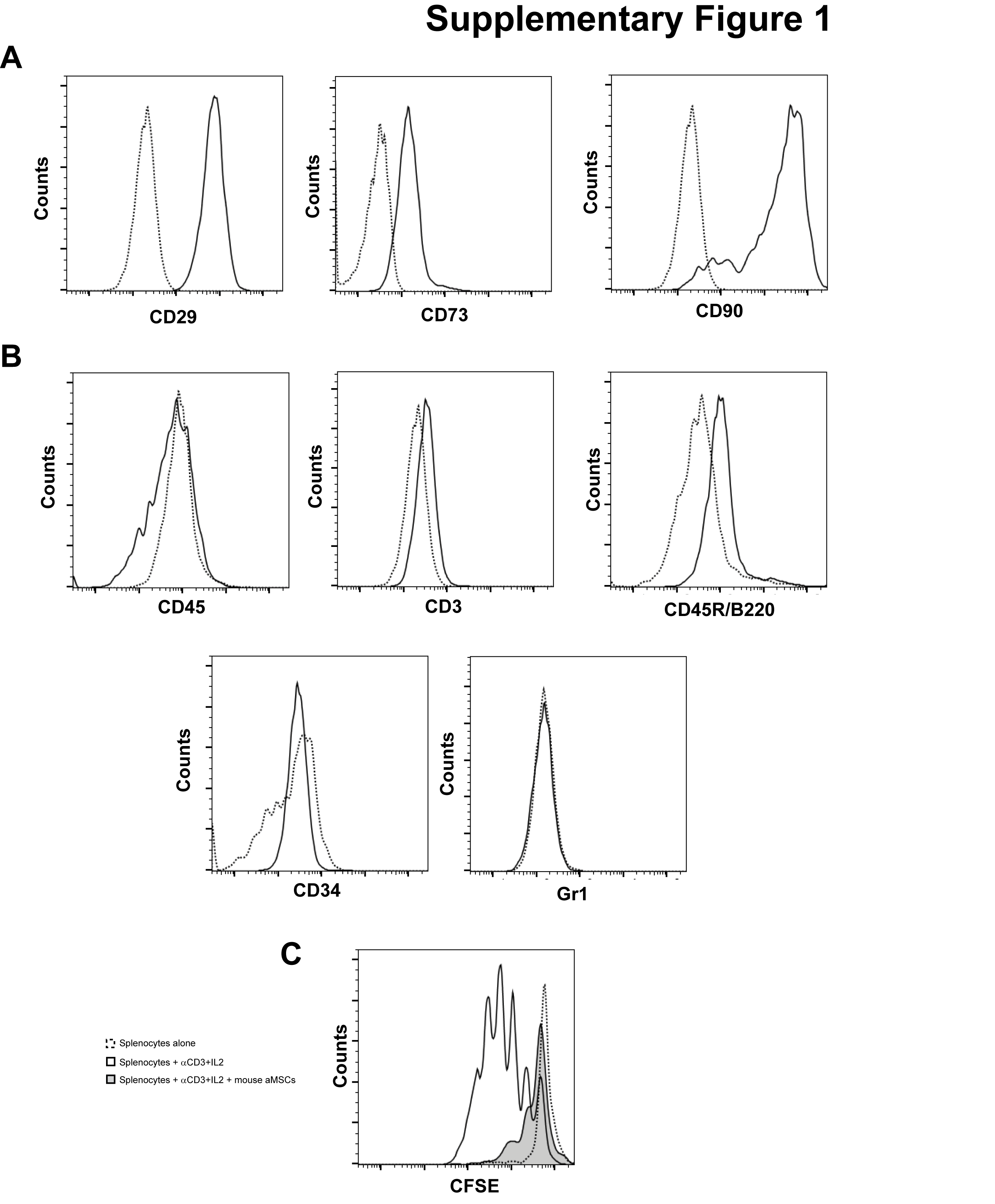

Supplement: Supplementary file 1 [file Image_1.tif]

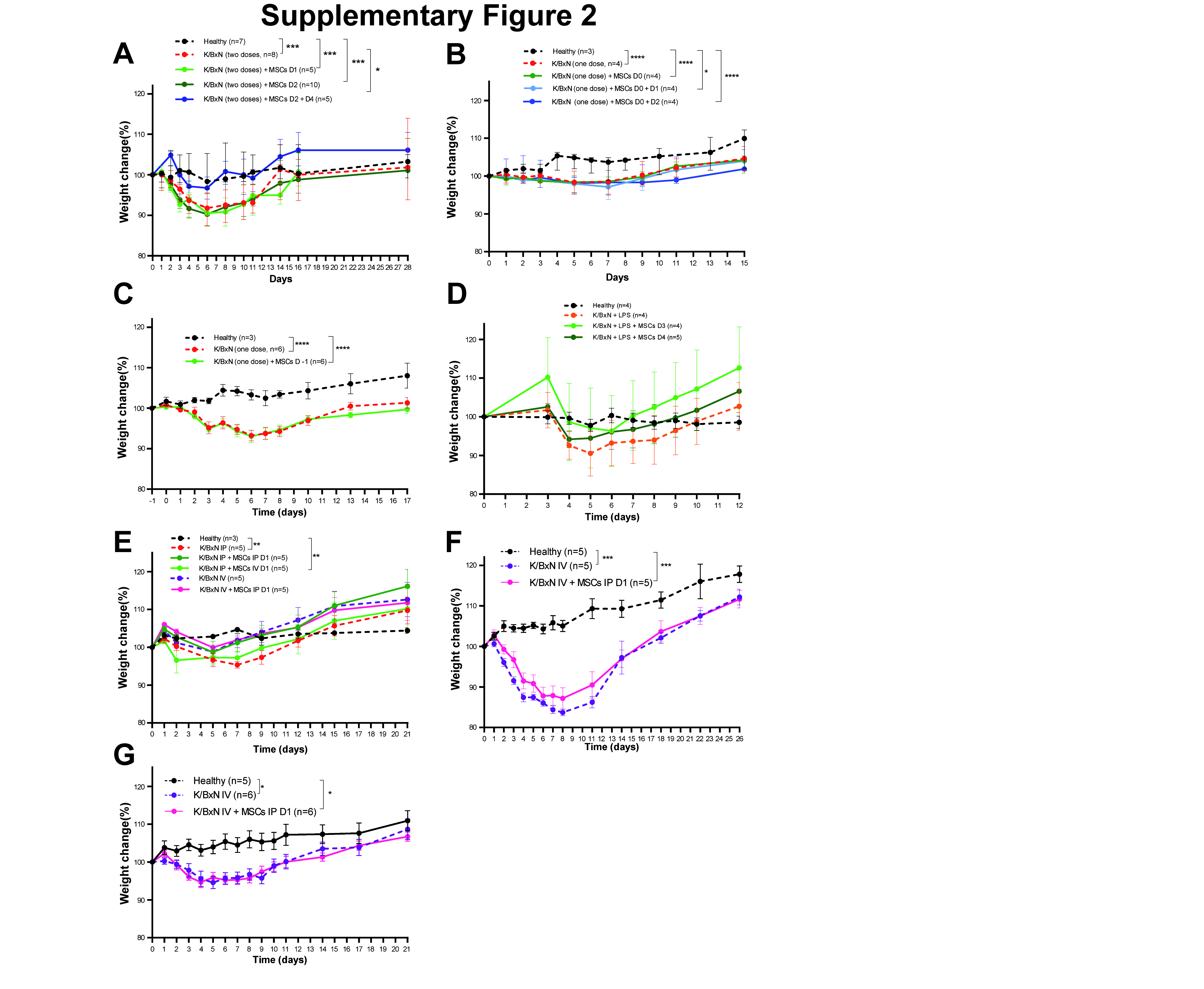

Supplement: Supplementary file 2 [file Image_2.tif]

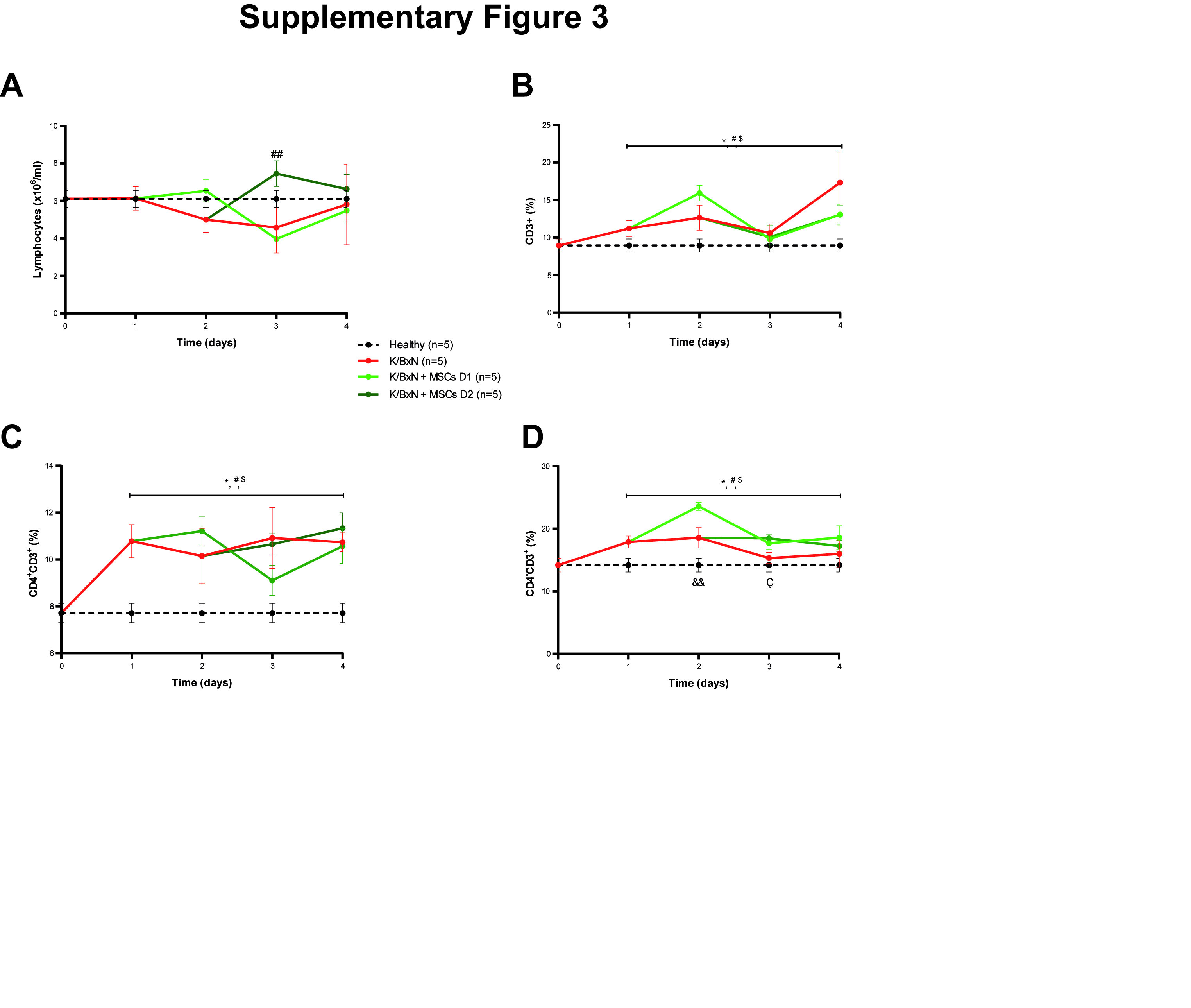

Supplement: Supplementary file 3 [file Image_3.tif]
